# Perspectives on Personalized Treatment in Difficult-to-Treat Depression: A Case Report

**DOI:** 10.1155/crps/5538951

**Published:** 2025-05-06

**Authors:** Walter Paganin, Sabrina Signorini

**Affiliations:** ^1^Doctoral School in Neuroscience, University of Tor Vergata, Rome, Italy; ^2^StudioPsicologiaSignorini, Guidonia, Rome, Italy

**Keywords:** difficult-to-treat depression, multidisciplinary approach to depression, patient and family education, personalized treatment, psychotherapy

## Abstract

**Introduction:** This case highlights the unique challenge of difficult-to-treat depression (DTD), a complex condition that distinguishes itself from treatment-resistant depression (TRD) due to its multifaceted nature. DTD is characterized by comorbidities, childhood trauma, symptomatic variability, personal history, substance use, and adherence issues, presenting a significant clinical challenge. Unlike TRD, typically defined by the failure of at least two adequate pharmacological treatments, DTD requires a more comprehensive approach. Recent literature supports a multidisciplinary treatment strategy as the most effective in managing DTD. The patient's main concerns and important clinical findings: We present the case of a 63-year-old female patient with a long-standing history of unresolved depressive disorder despite multiple pharmacological treatments. Her primary concerns included severe anhedonia, persistent suicidal ideation, and impaired personal and familial functioning. The patient's history included prior failed treatments, highlighting the complexity of her case. Primary diagnoses, interventions, and outcomes: The patient was diagnosed with DTD. A personalized treatment plan was implemented, integrating a clearly defined multidisciplinary approach: pharmacotherapy, psychotherapy (with a focus on grief and trauma processing), and psychosocial support, including active family involvement through psychoeducation sessions. Neurostimulation techniques were discussed as a potential alternative but were not applied in this case. Over time, the patient demonstrated significant improvement, with a reduction in depressive symptoms, resolution of suicidal ideation, and enhanced personal and familial functioning.

**Conclusion:** This case underscores the need for a personalized treatment approach for DTD that goes beyond pharmacotherapy to include psychotherapy, psychosocial support, and alternative options, such as neurostimulation when indicated. Active involvement of patients and their families is crucial, as evidenced by improvements in clinical and functional scores. Continuous monitoring and treatment adjustments based on objective measures (e.g., HRS-D, GAF, DTDQ) further optimize outcomes. The case exemplifies how an integrated treatment strategy can address the complexities of DTD, leading to long-term recovery and improved quality of life. The key takeaway is that managing DTD requires a comprehensive and individualized approach.

## 1. Introduction

**Figure 1:**
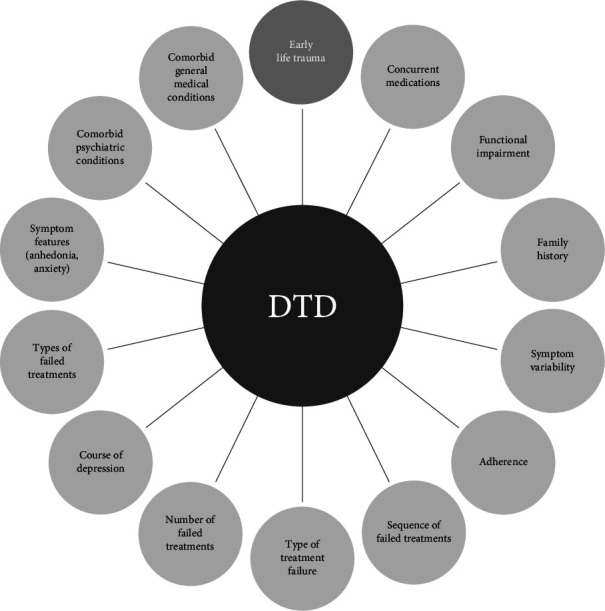
(Adapted from Rush AJ et al. clinical research challenges posed by difficult-to-treat depression. 2022).

The term difficult-to-treat depression (DTD) was historically used interchangeably with treatment-resistant depression (TRD) [1], leading to confusion among clinicians and complicating the understanding of TRD's precise definition [2]. However, in recent years, DTD has acquired its own taxonomic and conceptual specificity, largely due to the works of Rush, McAllister-Williams, Vieta, and others. In an international expert consensus statement, they proposed the DTD model based on a set of prognostic factors related to the patient, the disorder itself, and the treatments involved [3]. This conceptual and clinical evolution has shifted the focus from merely achieving a pharmacological response to a broader understanding of depression, the patient, and their quality of life. The emphasis now lies on optimal symptom control and the adoption of personalized treatment programs tailored to the patient's clinical history, comorbidities, and individual needs [1]. This shift in perspective is redefining the approach to depression therapy. While TRD primarily focuses on the inadequate or absent response to pharmacological treatment, DTD emphasizes factors that negatively interfere with the course of depressive disorder, such as psychiatric and medical comorbidities, childhood trauma, symptom variability, particularly anhedonia and anxiety, substance abuse, failed therapeutic strategies, family history, and adherence issues [4]. The formal and structural characteristics of DTD are shaped by the co-occurrence of clinical and developmental conditions that negatively affect the severity of depressive psychopathology (see Figure 1). This new perspective, embracing psychosocial, biological, and interactive aspects, opens the door to an integrated model of therapeutic governance.

We present a case of DTD with various course specifiers, where pharmacological interventions yielded a limited response. The patient was treated with a personalized and integrated care plan in relation to the clinical manifestations presented.

## 2. Patient Information

*Sixty-Three-Year-Old Female:* ***Family history*** *: Positive for psychiatric disorders: the patient's mother had generalized anxiety disorder*, *her older sister had depressive disorder*, *and her younger sister had obsessive–compulsive disorder*. ***Medical history****: As a child*, *the patient suffered from loss of consciousness, initially diagnosed as absences from childhood epilepsy*, *but later also related to failure of the ductus arteriosus to close with related hemodynamic imbalance. This diagnostic doubt has never been clarified anamnestically*, *but these symptoms have influenced her interpersonal relationships*, *leading to an introverted personality*, *limited friendships*, *and discontinuation of higher education. She reports childhood traumatic experiences*, *particularly related to a strict and overprotective parenting style*, *partially attributed to her epilepsy*. ***Social history****: The patient completed middle school*, *has been married for over 40 years*, *and is the mother of two adult children* (*a 39-year-old son and a 41-year-old daughter*), *both independent with families of their own. She worked as a secretary in her husband's business until the time of her consultation*. ***Treatment history****: 11 years prior*, *the patient was followed by the local Mental Health Center for “anxiety with insomnia” and was treated for 6 months with Paroxetine drops* (*20 drops/day*), *Bromazepam drops* (*30 drops twice a day*), *and melatonin tablets* (*1 mg at night*). *She discontinued treatment after her anxiety symptoms subsided. The subsequent and progressive onset of depressive symptoms starting at age 59 led to recurrent depressive episodes*, *which did not respond satisfactorily to pharmacological treatments*, *with residual symptoms between episodes. Pharmacological treatments*, *including selective serotonin reuptake inhibitors* (*SSRIs*), *serotonin-norepinephrine reuptake inhibitors* (*SNRIs*), *benzodiazepines* (*BZP*), *pregabalin*, *and mood stabilizers*, *were prescribed for reactive depression following the loss of her mother in 2021 due to COVID-19. These treatments did not produce an adequate response*, *indicating treatment resistance*.

### 2.1. March 2021

*Grief dynamics and disorder development: At the first consultation*, *the patient described deep sorrow and anger over her mother's solitary death*, *blaming herself for not being present. She had lived with her mother's daily presence*, *which she considered essential*, *and the inability to say goodbye after receiving the news of her death by phone intensified the trauma of the loss*, *leaving her with an unbearable emptiness. Due to COVID-19 restrictions*, *she was not allowed to see her mother upon her arrival at the hospital*, *which further exacerbated her sense of isolation and despair. This experience led to a significant deterioration in her emotional and psychological state*, *with mood deflection*, *anxiety*, *asthenia*, *anhedonia*, *and social withdrawal. There was a progressive loss of the ability to perform normal daily activities and work*, *along with the development of sleep and eating disorders. The following medications were prescribed: Duloxetine capsules* (*60 mg/day*) *and Pregabalin capsules* (*75 mg/day*). *After 3 weeks without response*, *the treatment was adjusted to Duloxetine* (*90 mg/day*) *and Pregabalin* (*75 mg twice a day*), *and 2 weeks later*, *the dosage was increased to Duloxetine* (*120 mg/day*), *Pregabalin* (*75 mg twice a day*), *and Lorazepam* (*2 mg at night*). *After 2 months*, *the patient experienced a partial response but discontinued the treatment due to weight gain. In parallel with the pharmacological treatment*, *she attended supportive psychological therapy sessions*, *which she discontinued after 3 months*.

### 2.2. July 2021

*Despite experiencing a partial response to the treatments*, *the patient continued to suffer from persistent anxiety*, *insomnia*, *and weight gain. She decided to consult another specialist*, *who adjusted the therapy to Duloxetine capsules* (*60 mg/day*) *and Aripiprazole tablets* (*5 mg at night*) *to address her insomnia and compulsive eating*.

Given the patient's age (63 years), it is appropriate to consider this case in the clinical context of late-life depression (LLD), characterized by higher rates of comorbidity, which often require age-adapted therapeutic strategies [5], however in the context of a DTD hypothesis, some target symptoms must be addressed to prevent treatment dropout and require specific treatment [3]. In this patient's case, particular attention was given to anxiety and insomnia. The treatment outcome involves the active engagement of the patient in their care and the optimization of treatment adherence. The goal is to improve symptom control and manage side effects through pharmacological adjustments based on measured care procedures, restore daily functioning and quality of life, and prevent or at least mitigate symptomatic relapses or recurrences [6].

### 2.3. October 2021

*As the patient continued to exhibit a partial response to pharmacological therapy*, *she resumed treatment prescribed by the other specialist: Duloxetine capsules* (*90 mg/day*) *and Pregabalin capsules* (*75 mg twice a day*), *discontinuing Aripiprazole. However*, *the treatment was once again suspended at the end of the year due to poor compliance*.

### 2.4. January 2022

*After several months*, *the patient*, *who had voluntarily discontinued pharmacological treatments due to weight gain and excessive sedation without improvement in her depressive state*, *presented for a consultation due to a worsening of depressive symptoms around the anniversary of her mother's death. The clinical case was marked by significant mood deflection* (*HRS-D = 26*), *the appearance of nonspecific suicidal ideation without any plan*, *and occupational dysfunction. The patient had left her job several months earlier and was socially withdrawing* (*GAF = 40*). *Clinically*, *the picture was dominated by a significant reduction in mood with anhedonia*, *lack of initiative*, *asthenia*, *low self-esteem*, *suicidal thoughts*, *insomnia*, *and weight gain. The DTD Questionnaire (DTDQ) was administered during the psychiatric evaluation to provide a structured assessment of treatment failure history*, *comorbidities*, *and psychosocial stressors. The patient scored 90*, *supporting the DTD diagnosis. Repeated administration allowed for tracking of clinical evolution and response to treatment. She scored 90 on the DTDQ*, *indicating DTD. After undergoing several pharmacological treatments that never resulted in symptomatic remission, some discontinued due to poor compliance*, *others due to side effects* (*weight gain*), *the patient underwent a joint psychological and psychiatric evaluation. Considering the challenging nature of her clinical condition*, *a multidisciplinary intervention was agreed upon with the patient*.

The diagnosis of depression is always a clinical diagnosis that can be supported by blood tests, neuroimaging techniques, and psychometric tests and scales to confirm or rule out the diagnosis. In this case, we used the 24-item Hamilton Depression Rating Scale (HRS-D) [7] to assess the severity of depressive symptoms, and the Global Assessment of Functioning (GAF) to evaluate the individual's overall functioning in the areas of social, occupational, and psychological well-being [8]. This scale describes specific functional conditions, thus aiding in diagnosis and treatment planning. Additionally, the recent DTDQ was used, designed to identify patients with depression who may not respond adequately to standard treatments and present management challenges. The DTDQ evaluates the number of failed pharmacological treatments due to lack of efficacy or side effects and includes questions on comorbidities, stress, social support, childhood and adult traumatic experiences, financial situation, and social and occupational functioning [9]. These tools were instrumental in this case for diagnosis, monitoring, and assessing treatment effectiveness.

*During the clinical course*, *the patient's personal history*, *family context*, *and the role she has always played within her family of origin were explored. It became evident that her grief was complicated by a complex family relational system*, *including secrets*, *dependency dynamics*, *and childhood traumas related to the upbringing she received and her neurological condition. The emotional impact of these past traumas was addressed*, *helping the patient develop greater coping skills and therapeutic self-management. The family members were also involved in the therapeutic process through psychoeducation sessions to better understand the patient's needs and provide the necessary support*.

When DTD is suspected, a thorough analysis of the patient's clinical history is essential. This should include an examination of the number and sequence of attempted treatments, the types of therapies and instances of treatment failure, the family history, the patient's adherence to prescribed treatments, and the possible presence of childhood trauma [10]. Scientific literature emphasizes the critical role of childhood traumatic events in the development and complexity of depression. Their presence during childhood often contributes to the challenges of managing DTD by leading to the emergence of comorbid disorders, thereby complicating therapeutic intervention strategies [11]. Moreover, a history of childhood trauma can exacerbate the course of depression, influencing both the selection and effectiveness of therapeutic treatments [4]. Consequently, identifying childhood trauma and assessing the potential for resulting medical and psychiatric complications is fundamental in defining targeted therapeutic strategies for patients with DTD.

### 2.5. March 2022

*During psychotherapy sessions*, *the patient was encouraged to resume pharmacological treatment*, *which she had previously discontinued due to side effects and a reduced therapeutic response. Discussions surrounding family-related issues contributed to a gradual*, *albeit slow*, *awareness of her psychological experiences*.

DTD is not a binary condition, but rather exists along a continuum of therapeutic responses [1]. Its spectrum varies, including complete, partial, or absent responses, which shifts the therapeutic approach from a curative model focused on healing to one centered on chronic disease management. This approach emphasizes improving functionality and quality of life, aiming for optimal symptom control [12].

### 2.6. April 2022

*After consulting a new specialist*, *the patient was prescribed Paroxetine tablets* (*20 mg/day*) *and Alprazolam drops* (*12 drops twice a day*). *However*, *despite not experiencing any side effects*, *the patient discontinued this therapy*.

Several factors can complicate the treatment of major depression, leading to DTD. These include an insufficient initial response to pharmacological therapy, the risk of relapse even during treatment, the necessity of using multiple antidepressants, the tendency for the illness to become chronic, and the risk of suicidal behaviors [1]. DTD significantly impacts patients' quality of life, increasing the risk of disability and other complications. The therapeutic psychotherapeutic intervention in this case focused on addressing early life events with traumatic significance, of which the patient had not yet fully recognized the emotional impact. It also supported the patient in adhering to pharmacological treatment and managing side effects.

### 2.7. September 2022

*The multidisciplinary care team implemented an integrated intervention strategy to strengthen the therapeutic alliance*, *collaborating closely with the patient's primary care physician for longitudinal assessment and evidence-based adjustments to treatment protocols informed by clinical metrics* (*e.g*., *vital signs*, *laboratory parameters*) *and patient-reported outcomes*, *the consulting psychiatrist for psychopharmacological optimization, including dose titration*, *side-effect monitoring*, *and referrals to specialized facilities such as eating disorder clinics, and the clinical psychologist to address unresolved grief and trauma through targeted psychotherapeutic modalities, while implementing systemic psychoeducational strategies involving the patient's spouse to foster collaborative decision-making and interpersonal communication. This unified approach synergistically combined pharmacotherapy*, *trauma-informed psychotherapy*, *social reintegration initiatives*, *and family-centered interventions within a cohesive treatment framework designed to address the patient's multidimensional vulnerabilities and enhance functional recovery*.

The integration of these practices, with a focus on both personalizing the therapy and building a strong physician-therapist-patient relationship, can significantly improve clinical outcomes in such cases [10].

### 2.8. November 2022

*The therapy initiated by the Mental Health Center* (*CSM*) *the previous month*, *consisting of Venlafaxine capsules* (*75 mg/day*) *and Lamotrigine tablets* (*25 mg/day*), *was increased to Venlafaxine capsules* (*75 mg twice a day*), *Lamotrigine tablets* (*25 mg with weekly increases up to 100 mg/day*), *and Zolpidem tablets* (*10 mg at night*). *The patient began to reflect on her family dynamics and the “caretaker” role she had inherited and perpetuated. Therapy sessions included the need to process grief*, *not only for her mother but also for the loss of her salvific role towards her sisters. Over the next 3 months*, *the patient exhibited significant improvement. Her mood improved*, *anhedonia decreased*, *and she began to take more initiative*, *experiencing increased self-esteem. Suicidal ideation disappeared*, *and there was a marked recovery in personal and family functioning. Attention to childhood traumatic experiences and the active involvement of the patient and her family in the therapeutic process contributed to enhancing her quality of life and promoting long-term well-being*.

The treatment of patients with DTD requires a complex multidisciplinary approach that can benefit from the contribution of new personalized medicine tools. It is likely that the combination of psychological treatments with pharmacotherapy and neurostimulation therapies may improve recovery rates [13, 14]. Family involvement, as described in some studies [15], has proven effective in treating complex and severe forms of depression, as family members' behavior, along with stigma, strongly influences the outcome of the illness [16]. Personalized treatment, including drug selection based on the patient's genetic characteristics, may offer a better therapeutic response, and the use of genetic testing to identify the most suitable medications could reduce the trial-and-error period typical of antidepressant selection [17].

### 2.9. April 2023

*The ongoing therapeutic work aims to support the patient in processing her grief and reassessing her family roles*, *with the goal of breaking a cycle of repetitive suffering and promoting a path toward healing and personal autonomy. A significant breakthrough occurred with the inclusion of the patient's husband in the therapeutic process*, *which provided a different perspective on the family system and marital dynamics. This allowed for the addressing and clarification of many previously unrecognized aspects of their relationship and the extended family context. The collaboration with her husband and the acknowledgment of marital issues have been fundamental steps toward the patient's recovery and the restoration of family balance*.

Family involvement, particularly through psychoeducation and the direct participation of the patient's husband in therapy, facilitated improved communication, reduced emotional burden, and supported adherence to the therapeutic plan. These relational shifts were associated with enhanced patient insight, reduced symptoms, and improved family functioning, consistent with the literature indicating the relevance of family involvement in complex depression cases. The complexity of this case lies in the intertwining of grief, reactive depression, and dysfunctional family dynamics. Research identifies psychotherapy as an important treatment modality for DTD, although its effectiveness may vary depending on the individual characteristics of the patient. A review of the literature suggests that while combined therapy of psychotherapy and medication shows greater efficacy, psychotherapy alone remains a vital component of treatment, especially for those who do not respond adequately to pharmacotherapy alone [18]. The understanding and treatment of DTD must be personalized and intensive, tailored to the complexities presented by each patient, to improve not only symptoms but also overall quality of life.

### 2.10. October 2023

*As part of an integrated treatment plan with social interventions*, *the patient was advised to join a gym*, *which she continues to this day*, *resulting in weight reduction. Additionally*, *she enrolled in a theater course*, *where she participated in an experiential “emotional theater” program*, *leading to partial resocialization. Neurostimulation treatments were not undertaken*, *as per the patient's choice*.

In addition to symptom control, interventions in DTD can aim to improve other factors, such as managing concurrent medical and psychiatric conditions, minimizing the therapeutic burden, enhancing daily functioning, improving quality of life or overall mental and physical well-being, mitigating symptom worsening, or otherwise reducing mood instability [6, 12].

### 2.11. December 2023

*Despite a new decline in mood*, *accompanied by anxiety and insomnia*, *the patient continued psychotherapy and did not discontinue pharmacological therapy. She was able to celebrate the holiday season with her family*, *with weekly clinical check-ups conducted during this period*, *and was supported in maintaining treatment adherence. Adjustments to the pharmacological therapy*, *based on the patient's individual response*, *included Venlafaxine* (*75 mg/day*) *and Lamotrigine* (*100 mg/day*). *These adjustments were crucial for maintaining psychological and clinical stability*, *reinforcing the psychotherapeutic practice*, *and processing the emotional impact of past traumas*, *thereby enhancing the patient's coping skills and therapeutic self-management*.

The goal of treatment in this case shifts from remission to optimal symptom control and improving quality of life. Therapeutic decisions must consider the patient's opinions in agreement with the medical team, through personalized interventions that “deconstruct” the factors contributing to the depression [19]. All possible therapeutic options should be examined with the aim of optimizing outcomes, especially when symptomatic remission is unlikely [3].

### 2.12. February 2024

*During the treatment*, *weekly clinical check-ups were conducted*, *and the patient was supported in managing side effects and maintaining treatment adherence. She continues to follow both pharmacological and psychotherapeutic therapies*, *while maintaining the resocialization activities that were recommended. Currently*, *the patient is taking Venlafaxine tablets* (*75 mg/day*) *and Lamotrigine tablets* (*100 mg/day*), *showing sufficient psychological stability with the disappearance of suicidal ideation and the restoration of personal and family functioning. Clinical outcomes were assessed using the Hamilton Rating Scale for Depression*, *GAF*, *and DTDQ at multiple time points* (*January 2022*, *November 2022*, *February 2024*). *Scores improved from HRS-D = 26 to HRS-D = 10, GAF increased from 40 to 75, and DTDQ decreased from 90 to 44. These measures provided objective data to support clinical improvement*, *guide therapy adjustments*, *and validate patient-reported outcomes*.

**Figure 2:**
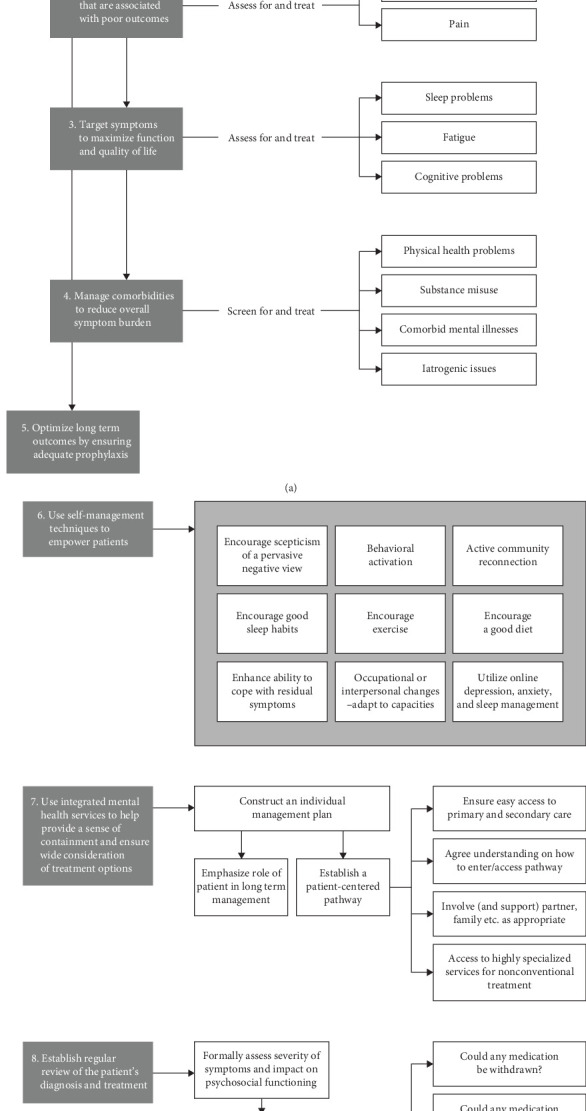
(Adapted from McAllister-Williams, Arango, Blier et al., the identification, assessment, and management of difficult-to-treat depression: An international consensus statement 2020).

Constant monitoring and the adjustment of therapy based on the individual response are crucial for achieving good psychological and clinical stability, as demonstrated in this case [10]. It illustrates how a multidisciplinary approach, combining pharmacological therapy with psychotherapeutic and social interventions aimed at restoring autonomy and self-esteem, can be effective in the treatment of DTD. Psychiatrists generally tend to focus primarily on alleviating depressive symptoms; however, in cases of DTD, seeking concordance between the physician and patient is an important element in defining the treatment plan [20]. Treatment stratification is based on a thorough understanding of the disorder's pathogenesis and requires continuous adaptation to individual needs (see Figure 2, Table 1).

## 3. Discussion

This clinical case may offer insights to inform reflections on clinical practice, particularly in managing patients with DTD. However, further research and evidence are required before these insights can influence treatment guidelines. The detailed description of an integrated, multidisciplinary approach provides a relevant perspective for managing patients with similar DTD-related challenges. The personalized treatment outlined here may encourage clinicians to prioritize individual patient needs alongside standardized protocols. This case is notable for the coexistence of TRD in later life, a history of childhood trauma, pathological mourning, and significant impairments in social and family functioning. The persistence of anhedonia and suicidal ideation despite multiple interventions, combined with therapeutic discontinuity, represents typical elements characterizing the clinical profile of DTD. Personalization of treatment, which takes into account specific factors such as comorbidities, family history, traumatic experiences, and treatment adherence, along with psychosocial support and the integration of family members into the therapeutic process, could become a common practice [22]. This may lead to greater emphasis on nonpharmacological components in the management of DTD in future guidelines. The evident benefits derived from educating and involving family members in treatment could encourage a more explicit recommendation of these elements as fundamental aspects of DTD care. Family training and support may receive increased attention, helping to improve not only the patient's quality of life but also that of caregivers, while the use of tools, such as the DTDQ may contribute to monitoring the disorder's progression [9]. Although neurostimulation techniques were discussed with the patient, they were not implemented in this case. Nevertheless, they remain valid options in cases of pharmacological failure and should be considered within a personalized care framework, when clinically appropriate. The management of DTD demands a structured, patient-centered framework that integrates pharmacological, psychological, and social domains. Building on the outcomes observed in this case, we propose a stepwise algorithm (Table 2) to guide clinicians in addressing the multifactorial nature of DTD. This algorithm emphasizes:

1. Early identification of psychosocial drivers (e.g., trauma, family dynamics).
2. Tailored interventions combining evidence-based pharmacotherapy, trauma-focused psychotherapy, and community reintegration.
3. Continuous adaptation of treatment based on objective metrics through standardized tools (HRS-D, GAF, and DTDQ) and patient-reported outcomes.

This clinical case not only provides a concrete model of success in the treatment of DTD but also opens new avenues for research and clinical practice, suggesting the importance of a holistic, personalized, and carefully monitored strategy for addressing this complex and challenging condition.

## 4. Conclusions

The conclusions from this clinical case on the management of DTD emphasize the importance of a targeted and personalized approach in treating this complex condition. In summary:

1. **Distinction between DTD and TRD**: The distinction between DTD and TRD, in addition to the theoretical definition, must occur in clinical practice through the analysis of factors such as symptomatic variability (e.g., persistent anhedonia), the presence of early traumatic events, psychiatric family history, and difficulties in adhering to treatments despite an adequate partial response. In the case presented, the combined approach integrated: (1) pharmacotherapy progressively adapted to the patient's tolerance and response; (2) psychotherapy focused on trauma and mourning; and (3) psychosocial interventions, including family psychoeducation. The synergy between these therapeutic levels allowed functional recovery and reduction of depressive symptoms, offering a reference model for other complex cases of DTD. This broader conceptual framework allows for a more comprehensive evaluation and management of patients.
2. **Multidisciplinary approach**: Effective treatment requires the integration of various therapeutic strategies, including pharmacological interventions, psychotherapies, psychosocial support, and somatic therapies, all tailored to the patient's specific needs and clinical history.
3. **Active involvement of patients and families**: The participation of both the patient and their family in the therapeutic decision-making process is crucial to optimizing outcomes. Psychoeducation and family support play a key role in improving clinical outcomes.
4. **Continuous monitoring**: Managing DTD requires constant monitoring of symptoms and treatment side effects, with therapeutic adjustments made based on the patient's response and needs.
5. **Awareness and training of healthcare professionals**: Promoting awareness among healthcare professionals of the multidisciplinary and psychosocial factors involved in DTD is essential. Continuous education is fundamental to ensuring informed and effective treatment.

These findings highlight the need for a comprehensive and adaptive treatment framework for DTD, taking into account its heterogeneous clinical presentation. The integration of psychosocial interventions and structured rehabilitation programs into treatment strategies can promote the restoration of autonomy and self-esteem, thus improving overall quality of life. Furthermore, family involvement is crucial to cultivate a nurturing environment that supports lasting recovery. Future research should prioritize investigations into the optimal integration of pharmacological, psychotherapeutic, and even neurostimulation modalities to address the multiple challenges inherent in DTD.

## Figures and Tables

**Table 1:** Multidimensional strategies for DTD.

1. Basic treatments and conventional and unconventional interventions
The first level of intervention should focus on the use of conventional treatments, which include first-line medications, targeted psychotherapies, and neurostimulation techniques. Antidepressants represent a cornerstone of pharmacotherapy, with dosage adjustments, switching medications, and augmentation strategies to be considered in response to the patient's reactivity [21]. Neurostimulation, such as electroconvulsive therapy (ECT), can offer an alternative or complement to pharmacological therapy for patients who do not adequately respond to or cannot tolerate the side effects of medications [2].
2. Addressing target symptoms and inadequate responses
Beyond basic treatments, it is essential to identify and target specific symptoms that persist despite the application of standard therapeutic protocols. These “target” symptoms, such as anxiety and pain, may require additional interventions, including the use of unconventional treatments. The combination of novel medications, integration of psychotherapy, and neurostimulation may be considered for these more complex cases [3].
3. Impact of symptoms on quality of Life
Particular attention must be given to symptoms that significantly impact the patient's quality of life, such as sleep disturbances, fatigue, and cognitive difficulties. Accurate diagnosis and targeted treatment of these symptoms are essential to improve the patient's overall well-being and daily functioning [2].
4. Management of comorbidities
The comorbidity of medical and psychiatric conditions, substance abuse, and iatrogenic phenomena represents significant clinical challenges in the management of mood disorders. A holistic assessment and an integrated treatment plan are necessary to effectively address these complicating factors [2–4].
5. Long-term perspectives and relapse prevention
It is imperative to optimize long-term outcomes through proactive management and appropriate prophylaxis. This may include patient education, regular monitoring, adjustment of therapeutic strategies to prevent relapse, and ongoing attention to the patient's overall health condition [6].
6. Promotion of self-management
A key element in the long-term treatment of mood disorders is patient empowerment through self-management techniques. It is essential to encourage patients to develop a critical perspective towards pervasive negative thoughts, promote healthy sleep habits, and strengthen their ability to manage residual symptoms. Behavioral activation, active community engagement, balanced nutrition, and physical exercise are all interventions that support self-management. Additionally, the use of online resources for managing depression, anxiety, and sleep can be a valuable support [3, 4, 7].
7. Integrated mental health services
To effectively address mood disorders, it is essential to utilize integrated mental health services that help contain the disorder and ensure comprehensive consideration of treatment options. Developing an individualized management plan, which emphasizes the patient's active role in the long term and establishes a patient-centered pathway, is fundamental. Furthermore, ensuring easy access to primary and secondary care and agreeing on a service access pathway, while involving and supporting family members, becomes crucial. Access to highly specialized services for unconventional treatments should be considered when indicated [7].
8. Regular patient assessment and review
A formal and regular assessment of symptom severity and their impact on the patient's psychosocial functioning is imperative. This should include reconsideration of the diagnosis and screening for comorbidities, as well as a review of predisposing, precipitating, and perpetuating factors. Additionally, it is important to consider whether a medication can be reduced or further optimized and whether new medication options (switch or augmentation) should be considered. A comprehensive review of management options, including medications, psychotherapies, neurostimulation, and psychosocial interventions, ensures that all possible therapeutic strategies are considered. Finally, evaluating whether referral to a highly specialized service is warranted may be a critical step for some patients [4, 9, 10].

**Table 2:** Generalized algorithm for managing DTD: A stepwise, multidimensional framework.

Phase	Key components	Interventions	Expected outcomes
Phase 1: Comprehensive assessment			

Clinical evaluation and diagnostic tools	- Clinical evaluation and history: Trauma (childhood/adult), treatment adherence, family dynamics, medical/psychiatric comorbidities. - Standardized scales: HRS-D (Hamilton Rating Scale for Depression), GAF (Global Assessment of Functioning), DTDQ (difficult-to-treat depression questionnaire).	- Administer scales to quantify symptom severity and functional impairment. - Conduct structured interviews to identify psychosocial stressors.	- Confirm DTD diagnosis (e.g., HRS-D ≥20, GAF ≤60, and DTDQ ≥70). - Differentiate DTD from TRD (focus on psychosocial/contextual factors).

Phase 2: Personalized treatment initiation			

Pharmacotherapy	- First-line: SSRIs/SNRIs. - Augmentation: Atypical antipsychotics, mood stabilizers. - Switching: Based on tolerability/side effects.	- Optimize doses, monitor for adverse effects (e.g., weight gain, sedation). - Address comorbidities (e.g., anxiety, insomnia).	- 20%–30% symptom reduction (HRS-D) within 4–6 weeks. - Improved adherence via patient education.

Psychotherapy	- Trauma-/grief-focused therapy (CBT, EMDR). - Systemic/family/multifamily therapy to address relational stressors.	- Weekly sessions for 12–16 weeks. - Involve family members in psychoeducation.	- Reduced emotional avoidance. - Enhanced coping skills and family support.

Psychosocial activation	- Social prescribing: Structured activities (exercise, art therapy, volunteering). - Peer support groups.	- Collaborate with community resources for reintegration.	- Gradual resumption of social/occupational roles.

Phase 3: Continuous monitoring and adaptation			

Metrics-driven follow-up	- Biweekly/Monthly: HRS-D, GAF, and DTDQ. - Side effect monitoring (e.g., metabolic parameters).	- Adjust pharmacotherapy (dose escalation, augmentation). - Intensify psychotherapy if stagnation.	- HRS-D ≤15 (partial remission). - GAF ≥65 (functional improvement).

Decision nodes	- Suboptimal response: Consider neurostimulation or novel agents (e.g., glutamatergic modulators). - Suicidal ideation: Crisis intervention protocols.	- Referral to specialized clinics if needed.	- Mitigation of high-risk behaviors. - Stabilization of acute symptoms.

Phase 4: Long-term maintenance and relapse prevention			

Consolidation	- Maintenance pharmacotherapy (minimum effective dose). - Booster psychotherapy sessions (monthly/quarterly).	- Address residual symptoms (e.g., anhedonia, cognitive deficits).	- HRS-D ≤10 (sustained remission). - DTDQ ≤50 (improved psychosocial resilience).

Relapse prevention	- Seasonal/anniversary monitoring (e.g., grief triggers). - Lifestyle interventions: Sleep hygiene, nutrition, and exercise.	- Develop a relapse prevention plan with patient/family.	- ≥80% treatment adherence. - Restoration of autonomy and quality of life.

## Data Availability

The data supporting the findings of this case report are available upon reasonable request from the corresponding author.
